# Young ERC Resuscitation Science Masterclass Journal Club article: “Estimating bias from withdrawal of life-sustaining treatment”

**DOI:** 10.1016/j.resplu.2026.101528

**Published:** 2026-10-10

**Authors:** Eva Santos-Miranda, Laetizia Elena Glassl, Chase Jay Canter, Mohammad Daoud, Aurora Magliocca

**Affiliations:** a Servicio Galego de Saúde, Galicia, Edif. Admtvo. San Lázaro, 15781 Santiago de Compostela, Spain; b Universidade de Santiago de Compostela, Campus Norte, Av. De Xoán XXII, s/n, 15782 Santiago de Compostela, Spain; c Medical University Vienna, Spitalgasse 23, 1090 Vienna, Austria; d ZOLL Cardiac Management Solutions, 121 Gamma Dr, Pittsburgh, PA 15238, USA; e Department of Emergency Services, Allegheny General Hospital, 320 E North Ave Ste 0, Pittsburgh, PA 15212, USA; f Ínönü University Liver Transplant Institute, Elazığ Yolu 15, KM, 44280 Battalgazi/Malatya, Türkiye; g Agenzia Regionale Emergenza Urgenza (AREU), Via Melchiorre Gioia 37, 20124 Milan, Italy; h Department of Pathophysiology and Transplantation, University of Milan, Via Francesco Sforza 35, 20122 Milan, Italy

**Figure:**
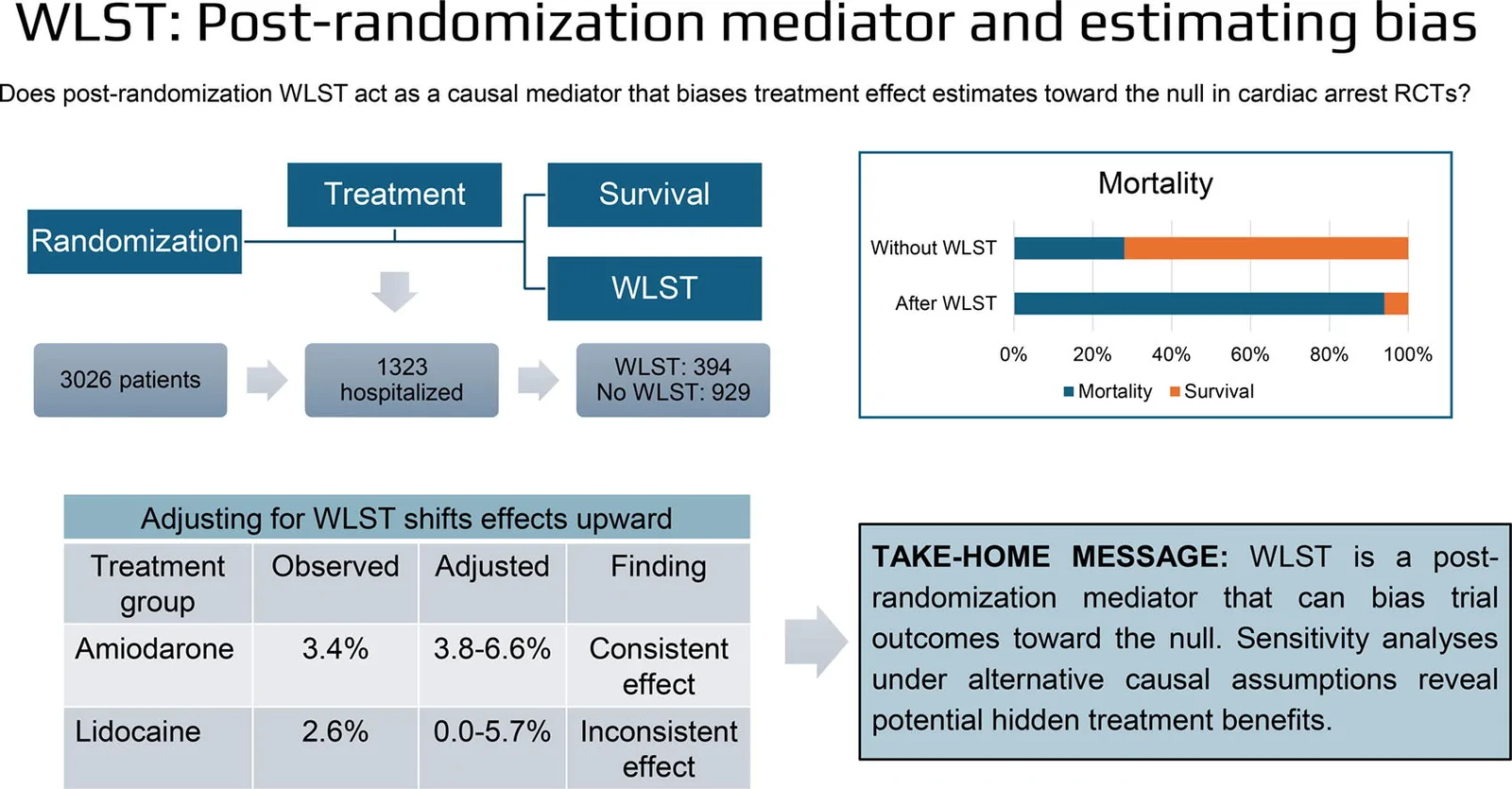

Published as part of the Young ERC Resuscitation Science Masterclass^1^ 2025/26, this journal club article discusses the 2025 critical care manuscript: “Estimating bias from withdrawal of life sustaining treatment in clinical trials”.^2^

## Which knowledge gap does this study try to fill out?

Callaway et al. address the lack of a systematic approach in evaluating whether withdrawal of life-sustaining treatment (WLST) acts as a post-randomization mediator capable of biasing outcomes in critical care trials.^2^ Mortality is the primary outcome in most cardiac arrest trials. Traditionally, deaths observed after resuscitation have been interpreted as a direct consequence of irreversible disease severity.^3^ A substantial proportion of deaths after cardiac arrest occur following WLST,^4^, ^5^, ^6^ suggesting that mortality may partly reflect clinical decision-making rather than biological disease progression alone.^7^, ^8^, ^9^

This issue is relevant in post-cardiac arrest care, where prognostication remains challenging despite advances in multimodal neuroprognostication.^10^ Current guidelines recommend delayed and multimodal assessment to minimize falsely pessimistic predictions, yet real-world adherence remains inconsistent.^11^ WLST practices vary considerably across institutions worldwide, with decisions often influenced by subjective clinical judgement, ethical considerations, cultural norms and local practice patterns.^12^

Despite these concerns, most randomized controlled trials (RCTs) on cardiac arrest do not systematically report WLST practices or evaluate their potential impact on treatment effect estimation.^5^, ^13^

## What was the design of the study?

In a methodological perspective article, Callaway et al. analyzed data from the randomized, double-blind, placebo-controlled Amiodarone, Lidocaine, or Placebo (ALPS)^14^ trial in patients with out-of-hospital cardiac arrest. In this proof-of-concept model, the authors examined whether WLST could have influenced or mediated the observed treatment effect. The study assessed if WLST met the criteria for being a causal mediator between treatment assignment and mortality. The authors conducted comparison of:

• Outcomes between WLST and non-WLST patients
• Baseline and clinical characteristics associated with WLST
• Characteristics of WLST patients across treatment groups
• Rates of WLST between treatment arms.

The authors then performed sensitivity analyses under different assumptions regarding the hypothetical outcomes of patients who died after WLST. These analyses included pessimistic and optimistic bounds, simple estimates, imputation models, inverse and augmented inverse probability weighting.

## What were the key findings of the study?

This study reframes WLST not merely as a limitation of critical care research, but as a measurable post-randomization intervention with potential causal implications.

Findings demonstrate a very strong association between WLST and mortality. Among hospitalized patients in the ALPS, mortality reached nearly 94% after WLST, compared to 28% in patients without WLST.

The authors observed that rates of WLST differed across randomized treatment groups, with both amiodarone (14%) and lidocaine (14.5%) groups showing higher odds of WLST compared to placebo (10.8%). Compared with placebo, the odds of WLST were higher in both the lidocaine (OR 1.41, 95% CI 1.08–1.83) and the amiodarone group (OR 1.35, 95% CI 1.03–1.76). This suggests that WLST may have mediated part of the observed treatment effect on survival.

The most important finding of the study emerged from the sensitivity analyses. Under the conventional interpretation of the trial, neither antiarrhythmic significantly improved survival. However, alternative assumptions regarding outcomes after WLST consistently yielded larger treatment-effect estimates, particularly for amiodarone.

## Are there any important methodological considerations to learn from the study?

Callaway et al. provide several important methodological lessons for future research. Randomization protects against baseline confounding but does not prevent bias arising from interventions or clinical decisions occurring after treatment allocation. When a post-randomization decision such as WLST is influenced by treatment assignment and is strongly associated with outcome, it may act as a mediator of the observed treatment effect. WLST should not simply be included as a conventional covariate in an adjusted analysis, as inappropriate adjustment for a mediator may itself introduce bias.^15^

The study illustrates that in the ALPS dataset, patients undergoing WLST are fundamentally different from those who continue receiving intensive care. Therefore, treating all deaths after WLST as inevitable may oversimplify outcome interpretation.

The authors should be commended for presenting multiple sensitivity analyses rather than claiming a single definitive estimate. This acknowledges the uncertainty surrounding counterfactual outcomes after WLST and provides plausible bounds for treatment effects. Such methodological transparency is one of the major strengths of the article. While randomization balances baseline confounders, post-randomization clinical dynamics—specifically mediator-outcome confounding and post-randomization selection—persist regarding decisions made in the ICU. Consequently, no statistical model can definitively establish counterfactual outcomes had life-sustaining therapy been continued.

Decisions to withdraw treatment may be influenced by prognostic assessment, patient and family preferences, institutional regulations, and cultural practices that may not be adequately represented in quantitative models. Cultural variation in end-of-life practices remains substantial, limiting external validity and complicating comparisons across international studies.^7^

Furthermore, in double-blinded trials such as ALPS, routine ICU WLST practices occur independently of treatment allocation, primarily diluting treatment differences and causing an attenuation of the effect (bias towards the null). In contrast, in unblinded trials, differential WLST decisions across arms could introduce systematic performance bias.

## What are the most important strengths and limitations of the study?

One of the major strengths is this articlés originality. While concerns regarding self-fulfilling prophecy after cardiac arrest have been discussed previously, few studies have attempted to systematically quantify how WLST may alter estimated treatment effects in RCTs.^16^, ^17^, ^18^ The authors translate this conceptual problem into a structured reproducible approach for determining whether WLST may influence treatment-effect estimates. Another strength is the use of the ALPS in which WLST was prospectively recorded.

Another strength is the cautious interpretation of the sensitivity analyses, which are presented to explore the uncertainty introduced by WLST rather than to argue the conclusions of the original RCT.

The study has important limitations. The analyses remain retrospective and exploratory. The true counterfactual outcomes of patients undergoing WLST can never be fully known. In addition, the assumptions underlying several statistical methods may not hold true in clinical practice. The study cannot determine whether individual WLST decisions were inappropriate or premature. Decisions regarding continuation of care after cardiac arrest are complex and frequently involve patient preferences, surrogate decision-making, ethical considerations, and resource limitations. The findings may not generalize across healthcare systems with different neuroprognostication practices and end-of-life cultures.

## How will the results affect your clinical practice?

This article has important implications for both bedside practice and interpretation of cardiac arrest research. From a research perspective, the study changes how clinicians may interpret mortality endpoints in cardiac arrest trials. Future appraisal of RCTs should include careful attention to:

• rates and timing of WLST
• neuroprognostication
• WLST protocols

The article does not suggest that WLST is inherently inappropriate. Instead, it emphasizes that mortality after cardiac arrest is not purely a biologically defined endpoint but may also reflect clinical, ethical, and cultural decisions.

## What do you see as the next steps in research?

Future resuscitation trials should prospectively and systematically collect data regarding WLST practices, including timing, indications, prognostic assessments, involvement of patient and family preferences. Standardized reporting of these variables could improve transparency and interpretation of outcomes. Further methodological work is needed to develop robust causal inference frameworks capable of addressing post-randomization mediators in critical care research. Approaches such as mediation analysis, target trial emulation, and advanced imputation strategies may become increasingly relevant.

Another priority is improving neuroprognostication itself. More accurate and better validated prognostic tools could reduce uncertainty and minimize variability in WLST decisions. Future studies should incorporate patient-centered outcomes beyond survival, including neurological recovery, quality of life, emotional wellbeing, and functional independence.

## Declaration of generative AI in scientific writing

AI was not utilized in the writing process.

## Declaration of transparency/disclosure

Given their role as an Associate Editor, Aurora Magliocca, had no involvement in the peer review of this article and had no access to information regarding its peer review. Full responsibility for the editorial process for this article was delegated to another journal editor.

## CRediT authorship contribution statement

**Eva Santos-Miranda:** Writing – original draft, Visualization, Data curation, Conceptualization. **Laetizia Elena Glassl:** Writing – review & editing, Writing – original draft, Visualization, Data curation, Conceptualization. **Chase Jay Canter:** Writing – original draft, Visualization, Data curation, Conceptualization. **Mohammad Daoud:** Data curation. **Aurora Magliocca:** Writing – review & editing, Supervision, Conceptualization.

## Declaration of competing interest

The authors Eva Santos-Miranda, Laetizia E. Glassl, Chase J. Canter and Mohammad Daoud declare that they have no known competing financial interests or personal relationships that could have appeared to influence the work reported in this paper.

The author Aurora Magliocca is a YERC Editor for this journal and was not involved in the editorial review or the decision to publish this article.
